# Socioeconomic Determinants of Diet Quality on Overweight and Obesity in Adults Aged 40–59 Years in Inner Mongolia: A Cross-Sectional Study

**DOI:** 10.3389/ijph.2021.1604107

**Published:** 2021-11-08

**Authors:** Yuenan Su, Sha Du, Min Yang, Jing Wu, Haiwen Lu, Xuemei Wang

**Affiliations:** ^1^ School of Public Health, Inner Mongolia Medical University, Hohhot, China; ^2^ National Center for Chronic and Non-Communicable Diseases Control and Prevention, Chinese Center for Disease Control and Prevention, Beijing, China; ^3^ Affiliated Hospital, Inner Mongolia Medical University, Hohhot, China

**Keywords:** socioeconomic status, dietary quality, alternate Mediterranean diet score, overweight and obesity, economic transition

## Abstract

**Objectives:** This study investigated the relationship of socioeconomic status (SES), diet quality and overweight and obesity in adults aged 40–59 years in Inner Mongolia.

**Methods:** This cross-sectional study was based on the survey of Chronic Disease and Nutrition Monitoring in Adults in Inner Mongolia in 2015. Diet quality was evaluated by the Alternate Mediterranean Diet score (aMeds). SES was measured by household annual income. Generalized estimating equations and path analysis were performed to determine the association of SES, diet quality and overweight and obesity.

**Results:** Among participants, 63.0% had overweight and obesity. In high SES group, 66.4% had overweight and obesity. Higher SES was associated with a higher risk of overweight and obesity (OR = 1.352, 95%CI: 1.020–1.793). And higher aMeds was associated with a lower risk of overweight and obesity (OR = 0.597, 95%CI: 0.419–0.851). There was a positive correlation between SES and the intake of red and processed meat (*r* = 0.132, *p* < 0.05). Higher intake of red and processed meat was associated with lower diet quality (*β* = −0.34). And lower diet quality was associated with a higher risk of overweight and obesity (*β* = −0.10).

**Conclusion:** In Inner Mongolia, during the period of economic transition, people aged 40–59 years in high SES had poor diet quality, which was related to a higher risk of overweight and obesity.

## Introduction

Overweight and obesity are risk factors for many chronic non-communicable diseases, such as cardiovascular disease, type 2 diabetes, and cancer [1, 2]. In recent years, the prevalence of overweight and obesity increased globally which accompanied by severe health damage and lower life quality among people [3]. 2017 Global Burden of Disease study showed that high body mass index (BMI) was the fourth risk factor leading to death [4]. Obesity had a great impact on middle-aged people [5], their lifestyle risk factors (smoking, unhealthy diet, or inadequate physical activity) could significantly increase the risk of chronic diseases in their old age [6–8]. The effective control of overweight and obesity among middle-aged people can significantly reduce the incidence of obesity-related chronic disease in their old age [9].

Socioeconomic status (SES) has an influence on overweight and obesity [10]. Generally, the association between SES and the risk of obesity is negative in high-income countries [11]. However, in some low- and middle-income countries (LMICs), people in high SES suffered a higher risk of overweight and obesity [12, 13]. Among the top 10 countries ranked by obese people number, eight countries were LMICs, including Brazil, China, India, etc., [14].

Diet quality plays an important role in the development of overweight and obesity in different SES participants. Some studies showed that high SES participants would adhere to healthy diet [15, 16]. A review of 29 meta-analyses demonstrated that higher adherence to the Mediterranean diet could reduce overall mortality, including diabetes, cardiovascular disease, and mortality of cancer [17]. However, some studies showed that some high SES individuals tended to choose some unhealthy dietary patterns, such as rich in animal fats or sugar which may relate to a higher risk of overweight and obesity [18, 19].

China is a middle-income country, and Inner Mongolia is an underdeveloped region in the north of the country. In recent decades, Inner Mongolia has been in a period of rapid economic development. In 2015, the prevalence of obesity was 18.1% [20], which was in a high level in China (11.3%) [21]. Our previous studies illustrated that people in Inner Mongolia deviated from healthy diet, and the prevalence of hypertension and diabetes were high in adults [22, 23]. Based on previous studies, we aimed to explore the relationship of SES, diet quality and overweight and obesity in adults aged 40–59 years in Inner Mongolia, for the purpose of contributing to the effective prevention and control of the overweight and obesity.

## Methods

### Design and Setting of the Study

This cross-sectional study was based on the survey of Chronic Disease and Nutrition Monitoring in Adults in Inner Mongolia in 2015. A multi-stage cluster random sampling method was used to obtain representative samples. A total of 841 participants aged 40–59 years were enrolled in this study. They were from eight monitoring sites including the urban, rural and pastoral areas in the eastern, central and western regions of Inner Mongolia. All participants provided written informed consent before the start of the investigation.

### Data Collection

All participants completed a questionnaire including information on sociodemographic characteristics, health-related behaviors, and diet. Height and weight were measured by trained investigators who followed standard protocols. 24-h dietary recall for three consecutive days was used to collect dietary data, all participants reviewed and described the types and amounts of all foods (including alcohol) they consumed.

### Measurements

#### Assessment of Diet Quality

The Alternate Mediterranean Diet score (aMeds) was used to evaluate participants’ adherence to the Mediterranean diet. The range of aMeds is 0–9. The higher the score, the better the diet quality. The aMeds components include whole grains, vegetables, fruits, legumes, nuts, fish, and the ratio of monounsaturated to saturated fat. Intake above the sex-specific median is scored as 1 point, others are scored as 0 points. Additionally, red and processed meat consumption below the median is scored as 1 point, others are scored as 0 points. For alcohol, 1 point is given if consumption between 5 and 15 g per day; others are assigned 0 points [24].

#### Assessment of Average Intake of Each Food Group

Based on the dietary data from 24-h dietary recall for three consecutive days, the average intake (g/day) of each food group was calculated among participants over 3 days [25].

#### Definition of Overweight and Obesity

BMI was calculated as weight in kilograms divided by height in meters squared, which was used as an indicator of overweight and obesity. BMI was divided into two categories: underweight or normal weight (<24 kg/m^2^) and overweight and obesity (≥24 kg/m^2^) [26].

#### Definition of SES

Household annual income was used as an indicator of SES, which was categorized as high SES [≥RMB 30,000 (US$4687)] or low SES [<RMB 30,000 (US$4687)] [27].

### Covariates

Sociodemographic characteristics included age, gender, ethnicity, marital status, residing location, and education level. Health-related behaviors were smoking status, drinking, and physical activity. The definition of variables was showed in Supplementary Table S1.

### Statistical Analysis

Continuous variables were expressed as means and standard deviations, and the *t*-test was used for two groups comparisons. Categorical variables were expressed as numbers and percentages, and the chi-square test was used to test significant differences between groups.

The aMeds was categorized into tertiles T1, T2, and T3. Higher scores indicated more compliance with the Alternate Mediterranean Diet, lower scores indicated more deviation. The dietary intake of participants and the recommended nutrient intake was compared [28].

Partial correlation analysis was used to examine the correlation between SES and aMeds, foods and nutrients. Generalized estimating equations were used to examine the association of SES and diet quality with the risk of overweight and obesity. Partial correlation analysis and generalized estimating equations were adjusted for age, gender, ethnicity, marital status, educational level, residing location, smoking status, drinking, physical activity. Path analysis was conducted to test the direct and indirect (through diet quality) effects of SES on the risk of overweight and obesity. The significance *α* was set to 0.05 and *p* ≤ 0.05 was considered significant. IBM SPSS Statistics 25.0 (IBM Corp, Armonk, NY, United States) and AMOS 25.0 were used for data analysis.

## Results

### Sociodemographic Characteristics of the Participants

A total of 841 participants aged 40–59 years were included in the present study. Just over half of the participants (51.2%) had high SES. Overall, 47.4% were men, 40.7% were urban, 80.2% were Han ethnicity, 14.4% were Mongolian ethnicity, 97.4% were married, and 40.1% had a primary school education or lower.

A total of 63.0% of the participants had overweight and obesity. Among those in high SES, 66.4% had overweight and obesity, which was higher than participants in low SES (59.5%). The prevalence of overweight and obesity among urban participants (68.1%) was higher than rural participants (59.5%) (Table 1).

**TABLE 1:** Sociodemographic characteristics of the adults aged 40–59. The survey of Chronic Disease and Nutrition Monitoring in Adults in Inner Mongolia, Inner Mongolia, 2015.

	Variables	Total n(%)	Overweight and obesity n(%)	*p*-Value
SES	Low	410 (48.8)	244 (59.5)	0.040*
	High	431 (51.2)	286 (66.4)	
Age (years)	40–44	166 (19.7)	105 (63.3)	0.544
	45–49	201 (23.9)	124 (61.7)	
	50–54	254 (30.2)	154 (60.6)	
	55–59	220 (26.2)	147 (66.8)	
Gender	Male	399 (47.4)	246 (61.7)	0.436
	Female	442 (52.6)	284 (64.3)	
Residing location	Urban	342 (40.7)	233 (68.1)	0.011*
	Rural	499 (59.3)	297 (59.5)	
Ethnicity	Han	690 (82.0)	431 (62.5)	0.288
	Mongolian	121 (14.4)	76 (62.8)	
	Other minority	30 (3.6)	23 (76.7)	
Marital status	Singled	6 (0.7)	4 (66.7)	0.982
	Married	819 (97.4)	516 (63.0)	
	Others	16 (1.9)	10 (62.5)	
Education level	Primary school and lower	337 (40.1)	217 (64.4)	0.473
	Junior high school	305 (36.3)	184 (60.3)	
	Senior high school and above	199 (23.6)	129 (64.8)	
Total		841 (100)	530 (63.0)	

*p < 0.05.

A total of 31.0% of the participants were in the aMeds T1 group. There were significant differences in overweight and obesity among aMeds and smoking status (*p* < 0.05). The highest prevalence of overweight and obesity (69.7%) was in the lowest aMeds group (T1) (Table 2).

**TABLE 2:** The proportion of overweight and obesity in different group of the Alternate Mediterranean Diet score and lifestyle factors. The survey of Chronic Disease and Nutrition Monitoring in Adults in Inner Mongolia, Inner Mongolia, 2015.

	Variables	Total n(%)	Overweight and obesity n(%)	*p*-Value
aMeds	T1	261 (31.0)	182 (69.7)	0.017*
	T2	296 (35.2)	183 (61.8)	
	T3	284 (33.8)	165 (58.1)	
Smoking status	Current smoker	270 (32.2)	155 (57.4)	0.011*
	Ex-smoker	43 ( 5.0)	34 (79.1)	
	Non-smoker	528 (62.8)	341 (64.6)	
Drinking	Excessive	113 (13.4)	67 (59.3)	0.378
	Never or Moderate	728 (86.6)	463 (63.6)	
Physical activity	None	65 ( 7.7)	41 (63.1)	0.893
	Inadequate	214 (25.4)	132 (61.7)	
	Sufficient	562 (66.9)	357 (63.5)	

*p < 0.05.

### Dietary Intake by SES

The average intake of different foods were showed in Table 3. The intake of cereals and tubers, legumes and nuts, vegetables, fruits, aquatic products, eggs, and dairy products were lower than the RNI (Table 3).

**TABLE 3:** The comparison of dietary intake of participants and the recommended nutrient intake. The survey of Chronic Disease and Nutrition Monitoring in Adults in Inner Mongolia, Inner Mongolia, 2015.

Foods (g/day)	RNI	Average intake ( $\bar{x}$ )		
		Total	Low SES	High SES
Cereals and tubers**	250–400	320.27	303.59	336.13
Vegetables	300–500	149.16↓	154.08↓	144.48↓
Fruits**	200–350	40.46↓	30.61↓	49.82↓
Red and processed meat**	40–75	86.46↑	73.68	98.60↑
Legumes and nuts*	25–35	30.66	26.53	34.59
Aquatic products	40–75	7.28↓	5.96↓	8.53↓
Eggs	40–50	17.18↓	15.33↓	18.94↓
Dairy products	≥300	31.80↓	30.88↓	32.68↓

*p < 0.05.

**p < 0.01.

RNI, recommended nutrient intake; ↑, Per capita intake is higher than RNI; ↓, Per capita intake is higher than RNI.

There were significant differences by SES level in the intake of cereals and tubers, fruits, red and processed meat, legumes and nuts (*p* < 0.05). The intake of cereals and tubers, fruits, red and processed meat, and legumes and nuts were higher in high SES group than in low SES (Table 3).

### Relationships Between SES and aMeds, Foods and Nutritions

The partial correlation analysis showed that there was no correlation between SES and aMeds. There were positive correlations between SES and the intake of fruits and of red and processed meat (*r* = 0.085 and *r* = 0.132, respectively; *p* < 0.05). SES was positively correlated with the intake of energy and protein (*r* = 0.097 and *r* = 0.187, respectively; *p* < 0.01) (Table 4).

**TABLE 4:** The partial correlation analysis among socioeconomic status and the Alternate Mediterranean Diet scores, foods and nutrients. The survey of Chronic Disease and Nutrition Monitoring in Adults in Inner Mongolia, Inner Mongolia, 2015.

Variables (g/day)		*r*
aMeds		0.013
Foods	Vegetables	−0.034
	Fruits	0.085*
	Nuts	0.060
	Whole grains	−0.015
	Legumes	0.046
	Fish	0.018
	Red and processed meat	0.132**
	Alcohol	−0.001
Nutrients	Energy (Kcal)	0.097**
	Carbohydrate (g/day)	0.043
	Protein (g/day)	0.187**
	Fat (g/day)	0.081

*p < 0.05.

**p < 0.01.

Note: adjusted for age, gender, ethnicity, marital status, educational level, residing location, smoking status, drinking, physical activity.

### Association of aMeds and SES with the Risk of Overweight and Obesity

In the generalized estimating equations, aMeds and SES was associated with the risk of overweight and obesity. Compared with those in low SES, participants in high SES had a higher risk of overweight and obesity [odds ratio (OR) = 1.352, 95% CI: 1.020–1.793]. Compared with those in low aMeds, participants in highest aMeds had a lower risk of overweight and obesity (OR = 0.597, 95% CI: 0.419–0.851) (Table 5).

**TABLE 5:** The relationship between the Alternate Mediterranean Diet score, socioeconomic status and overweight and obesity. The survey of Chronic Disease and Nutrition Monitoring in Adults in Inner Mongolia, Inner Mongolia, 2015.

	Variables	OR	95% CI
SES	Low	1.000	
	High	1.352*	(1.020,1.793)
aMeds	T1	1.000	
	T2	0.704	(0.494,1.002)
	T3	0.597**	(0.419,0.851)

*p < 0.05.

**p < 0.01.

Note: adjusted for age, gender, ethnicity, marital status, educational level, residing location, smoking status, drinking, physical activity.

### Path Analysis of the Association of SES and Diet Quality with Overweight and Obesity

**FIGURE 1:**
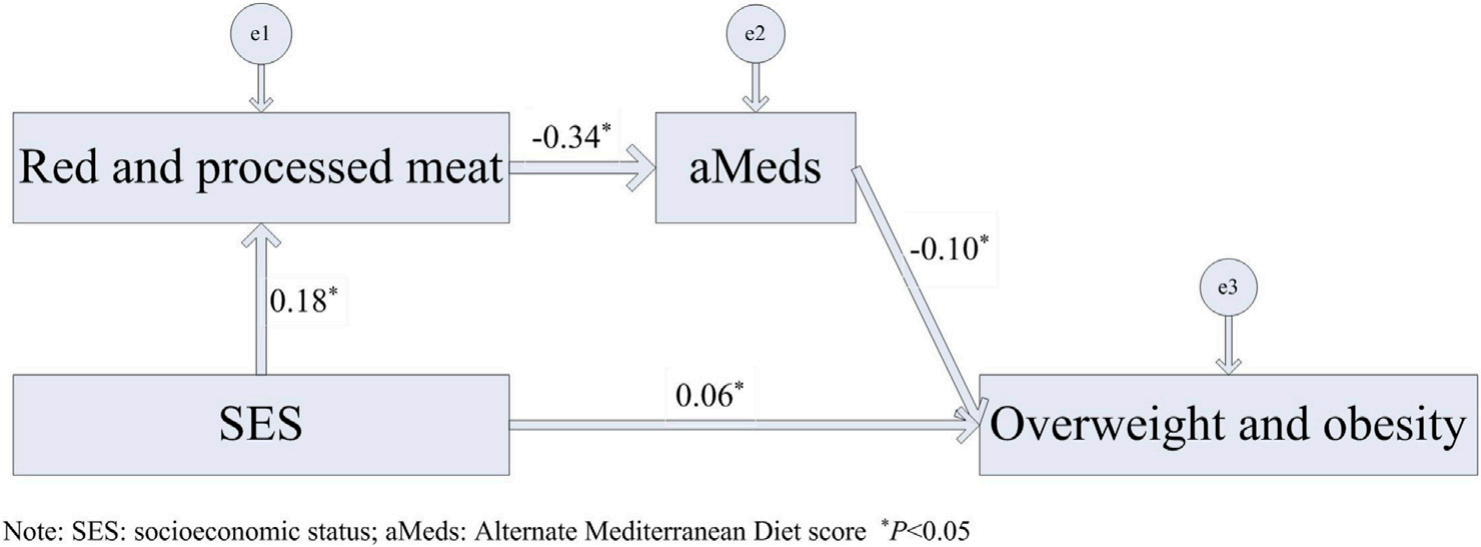
Path analysis of the associations of socioeconomic status and diet quality with overweight and obesity in adults aged 40–59 years. The survey of Chronic Disease and Nutrition Monitoring in Adults in Inner Mongolia, Inner Mongolia, 2015.

The path analysis showed that the direct effect of SES on the risk of overweight and obesity was significant (*β* = 0.06). More importantly, the indirect effect of SES on overweight and obesity was also significant, and it mainly related to the intake of red and processed meat (*β* = 0.18). Higher intake of red and processed meat was associated with lower diet quality (*β* = −0.34), and lower diet quality was associated with a higher risk of overweight and obesity (*β* = −0.10). Therefore, diet quality had a crucial mediating effect on the association between SES and the risk of overweight and obesity (Figure 1).

## Discussion

Inner Mongolia is an underdeveloped region in China with insufficient medical resources. Over the last 10 years, Inner Mongolia has been in a period of rapid economic development. In our study, 63.0% participants aged 40–59 years had overweight and obesity, which was in a high level in China (11.3%) [21]. Among the participants in high SES, 66.4% of had overweight and obesity. The participants deviated from a healthy diet, and 69.7% of the participants in low diet quality had overweight and obesity. In past 10 years, participants were facing increased dietary problems and the risk of overweight and obesity in Inner Mongolia.

### Comparisons with Other Studies

Previous evidence demonstrated that high SES was associated with better health outcomes [29–31]. However, with the rapid development of economic in LMICs, individuals in high SES suffered a higher risk of obesity compared with low SES [32]. In our study, high SES was associated with a higher risk of overweight and obesity, and the OR was 1.351. Similarly, previous studies showed positive correlations among SES level and obesity, diabetes, metabolic syndrome, and cardiovascular disease in LMICs [12, 33, 34]. A study covering 757,958 participants showed that 70–90% burden of diabetes, hypertension, and obesity was concentrated in high SES group [35].

Modifiable dietary characteristics are important explanatory factors for the association between SES and overweight and obesity. Some studies demonstrated that individuals in high SES adhered to relatively healthy dietary patterns [16, 36, 37]. In contrast, some individuals in high SES who were able to obtain excess food, tended to choose a high-fat and high-calorie diet during the economic transition in LMICs [18, 38]. Deviating from a healthy dietary pattern would result in an increase of overweight and obesity including their complications [39]. Similarly, our study also showed that diet quality had a significant mediating effect on the association between SES and the risk of overweight and obesity. Participants in high SES had a relatively high intake of red and processed meat, which resulted in poorer diet quality, leading to a higher risk of overweight and obesity. More importantly, the indirect effect of SES on overweight and obesity was also significant, working mainly through the intake of red and processed meat. Our result also showed that the intake of red and processed meat in all participants was higher than the RNI, especially in high SES participants. Energy intake of high SES participants was also higher than low SES. Excessive intake of red and processed meat led to excessive energy intake, which reflected poor diet quality and associated with a higher risk of obesity and diabetes [40, 41].

### Strengths and Limitations

This study was based on the survey of Chronic Disease and Nutrition Monitoring in Adults in Inner Mongolia in 2015 conducted in urban, agricultural, and pastoral areas. A multi-stage cluster random sampling method was used to obtain a representative sample of participants. However, this cross-sectional study could only show the association of SES and diet quality with the risk of overweight and obesity, but unable to demonstrate causality of them. Therefore, in the future, it is necessary to conduct more prospective studies on the complex effects of SES and diet quality on the risk of overweight and obesity among middle-aged people especially in LMICs.

### Conclusion

During the period of economic transition in Inner Mongolia, high SES participants aged 40–59 years had a relatively poor diet quality, which was related to a higher risk of overweight and obesity. Therefore, the health status of individuals in high SES should be given more attention, especially for middle-aged people in the relatively underdeveloped region in LMICs.
