# Supplementary material for: Socioeconomic Determinants of Diet Quality on Overweight and Obesity in Adults Aged 40–59 Years in Inner Mongolia: A Cross-Sectional Study
Source: Int J Public Health. 2021 Nov 8;66:1604107. doi: 10.3389/ijph.2021.1604107 (PMC8607545; doi:10.3389/ijph.2021.1604107)
Supplement: Supplementary file 1 [file Table1.docx]

Journal：

International Journal of Public Health

Article title：

Socioeconomic Determinants of Dietary Quality on Overweight and Obesity in Adults Aged 40-59 Years in Inner Mongolia: A Cross-sectional Study

Supplemental file 1: Table S1. Variables and coding in the analysis

Table S1 The definition of variables in the analysis

The survey of Chronic Disease and Nutrition Monitoring in Adults in Inner Mongolia, Inner Mongolia, 2015

| Variables | Categories | | |
| --- | --- | --- | --- |
| Overweight and obesity | No=0 | Yes=1 |  |
| Gender | Male=0 | Female=1 |  |
| Residing location | Urban=0 | Rural=1 |  |
| Ethnicity | Han=0 | Mongolian=1 | Other minority=2 |
| Marital status | Married=0 | Singled=1 | widowed/divorced=2 |
| Education level | Primary school and lower=0 | Junior high school=1 | Senior high school and above=2 |
| Smoking status | Current smoker=0 | Ex-smoker=1 | Non-smoker=2 |
| Drinking | Never or Moderate=0 | Excessive=1 |  |
| Physical activity | None=0 | Inadequate=1 | Sufficient=2 |
| aMeds | T1=0 | T2=1 | T3=2 |
| SES | Low=0 | High=1 |  |
